# A comparison of two common flight interception traps to survey tropical arthropods

**DOI:** 10.3897/zookeys.216.3332

**Published:** 2012-08-21

**Authors:** Greg P.A. Lamarre, Quentin Molto, Paul V.A. Fine, Christopher Baraloto

**Affiliations:** 1 Université Antilles-Guyane, UMR Ecologie des Forêts de Guyane, Campus agronomique de Kourou. Avenue de France. 97310, Kourou, French Guiana; 2 CIRAD, UMR Ecologie des Forêts de Guyane. Campus agronomique de Kourou. Avenue de France. 97310, Kourou, French Guiana; 3 INRA, UMR Ecologie des Forêts de Guyane, Campus agronomique de Kourou. Avenue de France. 97310, Kourou, French Guiana; 4 Department of Integrative Biology, 1005 Valley Life Sciences Bldg. #3140, University of California, Berkeley CA 94720; 5 Department of Biology, University of Florida, Gainesville, FL 32611

**Keywords:** flight interception trap, malaise trap, performance, sampling strategies, tropical forest, windowpane trap, French Guiana

## Abstract

Tropical forests are predicted to harbor most of the insect diversity on earth, but few studies have been conducted to characterize insect communities in tropical forests. One major limitation is the lack of consensus on methods for insect collection. Deciding which insect trap to use is an important consideration for ecologists and entomologists, yet to date few study has presented a quantitative comparison of the results generated by standardized methods in tropical insect communities. Here, we investigate the relative performance of two flight interception traps, the windowpane trap, and the more widely used malaise trap, across a broad gradient of lowland forest types in French Guiana. The windowpane trap consistently collected significantly more Coleoptera and Blattaria than the malaise trap, which proved most effective for Diptera, Hymenoptera, and Hemiptera. Orthoptera and Lepidoptera were not well represented using either trap, suggesting the need for additional methods such as bait traps and light traps. Our results of contrasting trap performance among insect orders underscore the need for complementary trapping strategies using multiple methods for community surveys in tropical forests.

## Introduction

Recent estimates suggest there are between 3 to 6 million arthropods species on Earth (Thomas 1990, Ødegaard 2000, Novotny et al. 2002, Hamilton et al. 2010), but these estimates remain a subject of debate because no more than 30% of tropical insects are currently described (Godfray et al. 1999). Tropical forests likely support most of the insect diversity on earth, but only a few studies have attempted to broadly sample insect communities in tropical forests. One reason that there remains little consensus regarding the total number of insect species is because there has been so little sampling in the Neotropics (Basset et al. 2005). Large scale and multi-protocol projects including IBISCA (Basset et al. 2007) and the ALAS project (Longino and Colwell 1997) have produced different sampling methodologies in different regions, including Central America (Basset et al. 2007), Australia (Stork et al. 1997; Kitching et al. 2001), and Africa (Missa et al. 2009). However, it is difficult to integrate data from the few existing studies because of a lack of standardized methods for insect sampling across locations and/or regions.

A massive sampling strategy of arthropods via an insecticidal fogging method is the technique most widely used in the tropics to study host specialization or the vertical stratification of arthropods on focal tree species (Erwin 1982, Basset 2001, Wilkie et al. 2010). However, this method is generally used for canopy surveys and very few studies have investigated understory insect communities, especially in the Amazon basin. Key among the understory trap methods are interception traps, including the Malaise trap (MT), which is considered one of the most popular sampling strategies by entomologists (Malaise 1937, Townes 1972,Southwood and Henderson 2000, Leather 2005, Fraser et al. 2008).

In this study, we introduce a modified version of the windowpane trap, which recently has become popular in French Guiana, and we investigate the relative performance of this alternative trap in comparison with the more conventional malaise trap. We present results of a standardized arthropod survey across different habitats representative of lowland forests in French Guiana in both wet and dry seasons to evaluate the relative performance of interception traps for different insect orders. We then discuss the implications for arthropod surveys in tropical rain forests.

## Methods

### Study sites

The study was conducted in two different regions of French Guiana: Laussat Conservation Area of French Guiana (05°28'N, 053°35'W, ~ 2600 mm annual precipitation) located in the west, and Petite Montagne Tortue (04°19'N, 052°14'W, ~ 3900 mm annual precipitation) located in the east. Climate in the region is driven by a seasonal alternation between a wet season (December to August) and a dry season (September to November). For each site, we conducted a long-term insect sampling campaign within permanent vegetation plots representing the three dominant tropical forest habitats in each region. We will compare trap performance among insect orders in French Guiana region that include common habitat types throughout the Amazon basin (Wittman et al. 2006, Baraloto et al. 2011): terra firme forests, flooded forests and white-sand forests.

Each plot is designed using modified Gentry methods of ten aggregate transects of 50 m subplot across a 2 ha area, with measures of soil and botanical descriptors (for plot details see Baraloto et al. 2011).

### Insect Sampling

**Figure 1.**
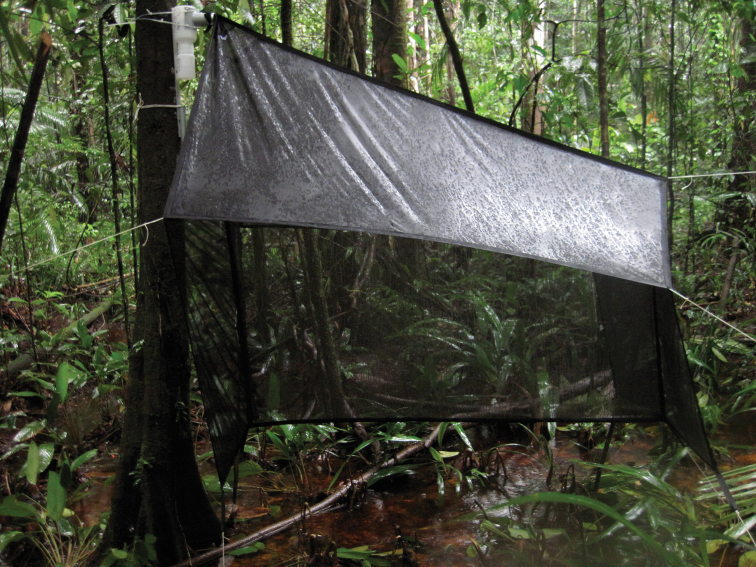
Picture of a malaise trap installed in a flooded forest of French Guiana (Lamarre G).

Malaise traps (MT) are designed to intercept insects flying through the understory, and they function by passively collecting the many insects that exhibit geotaxis and/or heliotaxis. Insects flying through the forest understory enter the central sheet of the MT, and fly upward until they fall into the collecting jar (Fig. 1). We used black malaise traps equipped with a transparent 500mL container filled with 96% alcohol. Our traps are a standard design constructed of lightweight black nylon mesh (EFE and GB Nets®, Bodmin, UK). More recently, the malaise trap has been modified to act as a flight interception trap using a mosquito net (as collecting surface) has become popular in tropical insect surveys (Barberena-Arias and Aide 2002, Chatzimanolis et al. 2004, Stork and Grimbacher 2006).

We introduce here a modified version of the windowpane trap (WT), which was originally based on suspended window frames (Southwood 1978, Chapman and Kinghorn 1995, Southwood and Henderson 2000). A large number of windowpane trap types have been developed based on this model (Springate and Basset 1996, Hill and Cermak 1997, Carrel 2002, Fielding 2003, Fayt et al. 2006, Bouget et al. 2008, Missa et al. 2009, Grimbacher and Stork 2009), but to date no standardized method has been widely accepted.

**Figure 2.**
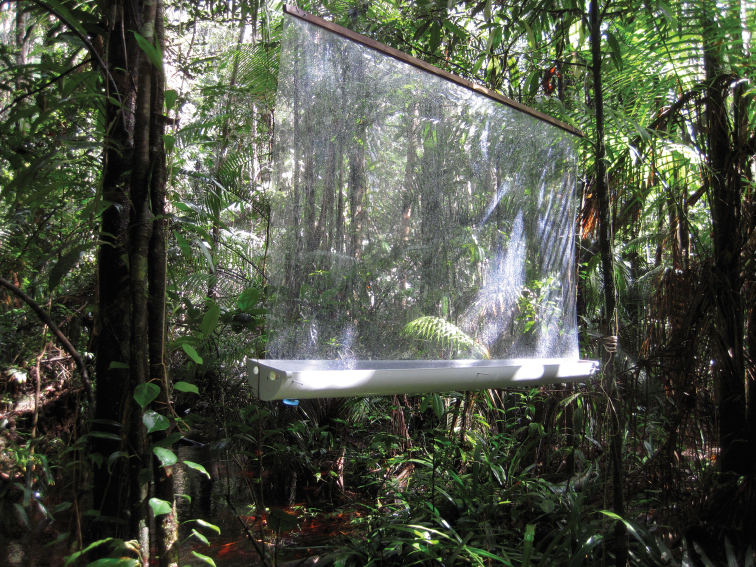
Picture of the modified windowpane trap described in this study (Lamarre G).

Here, we designed our WT to have a large transparent Plexiglas® pane that function as the interception surface (200 cm length; 130 cm width, 1 mm thick), in addition to a plastic rain gutter placed below the pane which functions as a collecting container (Fig. 2). In each lateral side of the gutter, two holes are drilled and filled with wire screening to evacuate rainwater. We inserted a collecting device beneath the gutter to empty insect collections from the trap. A mixture of 5L of water, 150 mL of detergent and 500 g of salt are used as killing and conservative agents, respectively. Fixed with two vertical ropes, a piece of wood is screwed into the Plexiglas pane to support the weight of the device. Using a metal screw (10 cm length), three holes are drilled in the bottom of the pane and attached to the gutter. The windowpane must be in the center of the gutter allowing for bi-directional capture of insects. We used a 5-liter water bottle top as a stopper. For each census, we opened the water bottle tap to empty the entire liquid/insect collection. A hole into the gutter has to be drilled with the exact same diameter of the stopper. We recommend the use of a powerful and hermetic glue to affix the stopper inside the gutter hole. Because it is made from lightweight plexiglass, our WT model is also easy to transport and to install. This type of insect trap can be built with low-cost materials. For example, in French Guiana (the most expensive country in the region), we estimate the cost per trap as 90 euros, whereas in Peru the materials to make the trap cost only 40 euros (prices verified in 2011 by the first author).

To compare the traps, we set up two pairs of each trap in each of six permanent plots of tropical forest, representing a total of 24 interception traps. Within each plot, pairs of MT-WT were installed in the same location in staggered rows at equal distance from each other (each traps are separated by at least 25 meters) on two representative sites with similar topography and canopy structure. Both WTs and MTs were attached and fixed to trees using cords and installed approximately two meters above the ground. A collection of each trap was made weekly for two census periods, each lasting three months; April to June and September to November 2010, respectively, corresponding to one dry and one wet season in French Guiana. We estimated trap performance as the sum of collected insect abundance across all orders using a standardized sampling protocol. We focused our study on seven well-studied arthropod orders: Blattaria, Coleoptera, Lepidoptera, Hemiptera, Hymenoptera, Orthoptera, and Diptera. Each collection was sorted to order and then to family level by the first author. Identification at species level is still pending in collaboration with taxonomic specialists.

### Statistical analyses

For each insect order, we modeled the number of captured insects with a quasi-Poisson Generalized Linear Model, which is appropriate for abundance and count data (Bolker et al. 2009). The explanatory variables included trap type (two levels), season (two levels), and plot (six levels). The first-order interactions were also included. An analysis of variance (ANOVA) was performed on each model. When the trap type variable had a significant effect (with a 5% critical probability), the trap type resulting in higher abundance was determined to be significantly more efficient than the other to capture the insects of the considered order. The statistical analysis was performed with R software 2.13.1 (R Core Team 2011).

## Results

**Figure 3.**
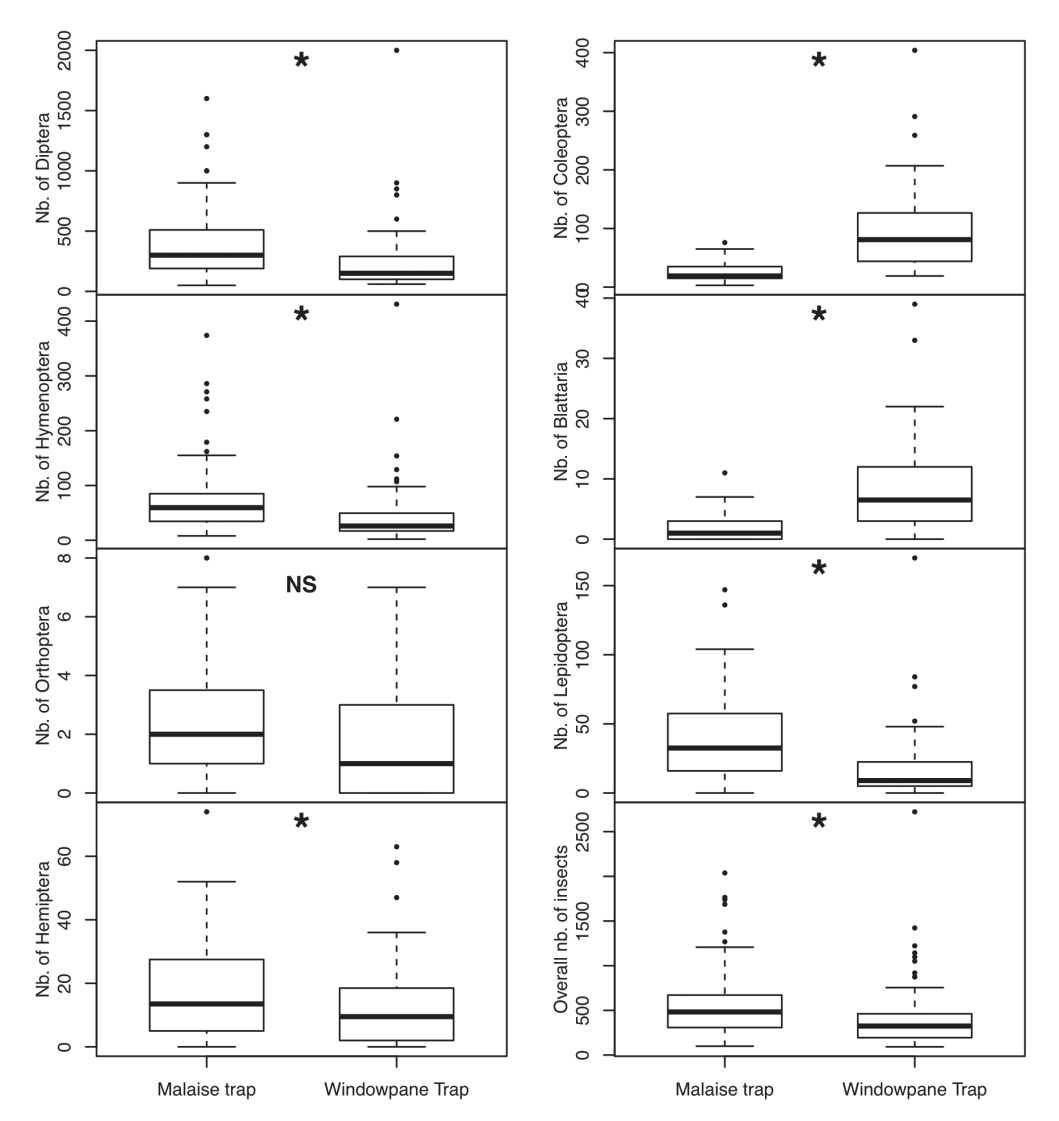
Box plot representing the relative abundance of the seven focal insect orders collected in each of the two traps. An asterisk above the bars represents significant differences between traps based on analysis of variance.

Overall, 71,822 individuals representing the seven focal insect orders were collected during the 6 month survey using the two types of entomological traps. We found consistent patterns in overall insect abundance between the two interception traps. Overall, MT caught more individual insects (41,292) than the new windowpane trap (30,530) (Fig. 3). We found that Diptera and Hymenoptera are caught more often by MT with nearly twice as many specimens as WT (F_(1,125)_= 24.9, P<0.001 for Diptera and, F_(1,125)_=2.95, P<0.001 for Hymenoptera). Our results show that the more efficient interception trap to sample Hemiptera is the malaise trap (F_(1,125)_=11.13, P=0.001). Beetles were more efficiently captured by WT than MT with nearly four times more beetle specimens captured using WT (F_(1,125)_=189,02, P<0.001). Blattaria yielded more specimens in our windowpane traps (F_(1,125)_=103,24, P<0.001) than our MTs. Lepidopterans were more effectively trapped by the MT (F_(1,125)_=62,60, P<0.001) than WT. We found no significant differences of Orthoptera abundance between the two interception traps (F_(1,125)_=3,1, P=0.08).

In summary, our results show that the most efficient trap to collect Diptera, Hymenoptera, Lepidoptera, Hemiptera and Orthoptera is the MT with significantly greater abundance than the WT. On the other hand, significantly more Coleoptera and Blattaria individuals were collected using the WT.

## Discussion

### Contrasting responses among insect orders

We report strong divergence in performance between interception traps among insect orders. Compared to WT, we found that MT captured significantly more small and lightweight insects which exhibit strong heliotropism and/or high mobility within the understory vegetation (i.e. Hemiptera, Diptera and Hymenoptera). In addition, we found that some insect orders, including Blattaria and Coleoptera, are likely to be collected most efficiently using WT because of differences in flight strength among orders. Our findings strongly suggest that beetles are best captured using WT, probably because WT can collect strong fliers like beetles more effectively than the MT.

### Diptera and Hymenoptera

Some authors argue that small-sized insects are likely to be blown by air currents into devices near the ground, which would explain the high density of flies when using the MT near ground level as we did (Kitching et al. 2004). This suggests that these groups of insect are more sensitive to the MT collecting surface representing an effective obstacle. Previous studies have found that malaise traps are highly effective for capturing Diptera and Hymenoptera (Ozanne 2005, Sääksjärvi et al. 2006, and Fraser et al. 2008) especially in the forest understorey (Ozanne 2005). We speculate that the low effectiveness of WT in capturing both Diptera and Hymenoptera is likely to be related to the poor point-to-point flight capabilities of these groups. High mobility in flight is probably related to the search of prey (or host plants), increasing the chance to be intercepted along a flight path by MT for Dipterans and Hymenopterans within the understory vegetation.

### Hemiptera

Most hemipterans are included within the sap sucker feeding guild (Moran and Southwood 1982); and hemipterans movement patterns in the forest understory are thought to be associated with the search for host plant (i.e. the availability of new leaves). Although hemipterans were more abundant in our MT than WT samples (Fig. 3), we note that overall both interception traps sampled a relatively low abundance of hemipterans. We therefore suggest that effective sampling of this order may require an additional complementary method such as light trapping (see Broadbent 1947; Hodkinson and Casson 1991).

### Coleoptera

Although similar types of WT have been employed before by entomologists, we adapted the design and made it larger than others have used in the past, expanding the width (i.e. interception surface) to at least twice the size of previous models (Hill and Cermak 1997, Bouget et al. 2008). We believe this may have made it even more effective at intercepting the flight path of Coleopterans. Furthermore, one difference between our WT model and other interception traps in general is that with our WT, beetles could be stunned by the window itself (in comparison to the soft cloth or plastic as interception material, see Springate and Basset 1996, Stork and Grimbacher 2006, Basset et al. 2007), leading to greater captures by WT compared to MT. Heavy beetles (i.e. Scarabaeidae, Cerambycidae, Passalidae etc.) are probably more likely to be stunned by the Plexiglas pane than lighter beetles. While others have noted that the use of heavy and bulky glass could damage some insect wings (Peck and Davis 1980), our model uses flexible and very thin Plexiglas that is less likely to damage insects. Our results strongly suggest that this alternative model of FIT could be an efficient alternative to capture beetles in tropical rainforest.

### Blattaria

Because they are not completely sclerotized, Blattaria are unlikely to be fatally stunned by the windowpane. We speculate that flying cockroaches are attracted to the device by the olfactory stimulus of other dead insects and/or the killing agent. Because they inhabit the litter at ground level, dead plant materials are thought to be the most important component of Blattarian diet (i.e. detritivory, see Bell et al. 2007). This order includes several other guilds as well, including wood feeders, scavengers, pollen and nectar feeders, although most of them generally feed on dead plant and animal material (Bell et al. 2007). Cockroaches are mostly associated with microhabitats within the understory. For this reason, if the goal is to sample Blattaria, we recommend the use of the windowpane trap installed close to the ground where the chance of capture is higher. However, specific insect traps installed within the litter could also be effective to capture cockroaches; we therefore also recommend the use of other types of traps, for example the pitfall trap (Sabu and Shiju 2008).

### Lepidoptera

Surprisingly, we caught a large number of adult moths and butterflies with both interception traps, which suggests that these insects fly through the forest understory with enough frequency to be effectively sampled by both MT and WT. Lepidopterans were more effectively trapped by the MT, which may be explained because they are more likely to be trapped within the malaise “tent”, flying upward towards either the sun (for butterflies) or the moon (for moths). However, we emphasize that the use of MT will only capture a small proportion of the Lepidopteran community, as many Lepidoptera species are not associated with understory vegetation. In our collection, the most abundant and diverse families were the Noctuidae for the moths and the Satyrinae for the butterflies. The latter are well known to fly close to the ground within the understorey (Braby and New 1988). We therefore recommend the use of bait trap and light trapping techniques as a complement to interception traps for butterfly and moth communities, respectively.

### Orthoptera

Very few data are available on tropical forest orthopterans, although there are a few studies on the grasshopper super-family Acridoidea in the canopy. In this group, population densities have been studied along a vertical gradient in French Guiana (Amédégnato 1997, 2003), with canopy grasshopper communities appearing to be richer than those in the understory. This trend could be explained by the very low abundance of orthopterans collected in both interception traps installed near the ground. As with Lepidoptera, we recommend the use of other type of traps for Orthoptera surveys such as light trapping techniques that exhibit very high efficiency in tropical forest surveys in Peru and French Guiana (G. Lamarre, unpubl. data).

### Perspectives for arthropod surveys in tropical forests

Our finding that so many coleopterans were captured by WT highlights the high level of flight activity of beetles, the most ecologically diverse group in the tropics, and strongly suggests that our model of WT should be used as an alternative method for future empirical studies contributing towards global as well as in areas that include gradients of anthropogenic disturbance. Furthermore, we recommend the use of our WT model to study ground beetles in forest microhabitats such as gaps, dead wood as well as anthropogenic gradient of perturbation. In French Guiana, some preliminary insect collections are showing very promising results with the placement of the WT up to 25 m above the ground within the forest canopy (S. Brûlé, pers. comm.). We therefore recommend the use of this interception trap for tropical arthropod surveys where coleopterans are the main targets, and we propose that it can nicely complement fogging methods for more comprehensive collections in the forest canopy.

To develop effective policies and management strategies in the context of escalating threats due to land use changes (Asner et al. 2010) and climate change in the Amazon basin (Malhi et al. 2008), we are in critical need of more complete descriptions of arthropod communities (May 2010). Indeed, arthropods represent an important indicator group to study future environmental changes in the tropics (Stork et al. 2003).

This study represents a first step towards a better understanding of how we should orient these sampling strategies. Our study clearly shows significant performance differences between two interception trap methods for the most common studied arthropods in tropical forests (Fig. 3). Among the seven groups on which we focused our study, at most three would be effectively sampled using a single trap method (MT for Dipterans, Hymenopterans and to a lesser extent Hemipterans), and only five would be well sampled using both methods (above in addition to WT for Coleopterans and to a lesser extent Blattaria), with two groups (Lepidoptera sensus largo and Orthoptera) requiring methods complementary to interception traps, such as light trapping and fruit traps. We therefore recommend that tropical entomological surveys should include a multiple-trapping-method approach rather than relying on a single trap type (Russo et al. 2011). We also advise the use of appropriate sampling techniques targeting focal insect groups (Basset et al. 2007). Our results clearly illustrate that there is no silver bullet for tropical arthropod sampling strategies.
